# Costs and cost-effectiveness analyses of mCARE strategies for promoting care seeking of maternal and newborn health services in rural Bangladesh

**DOI:** 10.1371/journal.pone.0223004

**Published:** 2019-10-01

**Authors:** Youngji Jo, Amnesty E. LeFevre, Katherine Healy, Neelu Singh, Kelsey Alland, Sucheta Mehra, Hasmot Ali, Saijuddin Shaikh, Rezawanul Haque, Parul Christian, Alain B. Labrique

**Affiliations:** 1 Bloomberg School of Public Health, Johns Hopkins University, Baltimore, Maryland, United States of America; 2 JiVitA Program, Johns Hopkins University, Gaibandha, Bangladesh; ESIC Medical College & PGIMSR, INDIA

## Abstract

**Objective:**

We examined the incremental cost-effectiveness between two mHealth programs, implemented from 2011 to 2015 in rural Bangladesh: (1) Comprehensive mCARE package as an intervention group and (2) Basic mCARE package as a control group.

**Methods:**

Both programs included a core package of census enumeration and pregnancy surveillance provided by an established cadre of digitally enabled community health workers (CHWs). In the comprehensive mCARE package, short message service (SMS) and home visit reminders were additionally sent to pregnant women (n = 610) and CHWs (n = 70) to promote the pregnant women’s care-seeking of essential maternal and newborn care services. Economic costs were assessed from a program perspective inclusive of development, start-up, and implementation phases. Effects were calculated as disability adjusted life years (DALYs) and the number of newborn deaths averted. For comparative purposes, we normalized our evaluation to estimate total costs and total newborn deaths averted per 1 million people in a community for both groups. Uncertainty was assessed using probabilistic sensitivity analyses with Monte Carlo simulation.

**Results:**

The addition of SMS and home visit reminders based on a mobile phone-facilitated pregnancy surveillance system was highly cost effective at a cost per DALY averted of $31 (95% uncertainty range: $19–81). The comprehensive mCARE program had at least 88% probability of being highly cost-effective as compared to the basic mCARE program based on the threshold of Bangladesh’s GDP per capita.

**Conclusion:**

mHealth strategies such as SMS and home visit reminders on a well-established pregnancy surveillance system may improve service utilization and program cost-effectiveness in low-resource settings.

## Introduction

Bangladesh, home to 165 million people, has a maternal mortality ratio of 176 per 100,000 live births and 74,000 neonatal deaths each year.[1] Almost 80% of the population live in rural areas with limited access to health care. Efforts to reduce maternal, newborn and child morbidity and mortality in Bangladesh have sought to bolster access to and utilization of health services through community health workers (CHWs)[2][3, 4] which has been a key strategy for providing preventive services and clinical care in the home with the broader aim of extending the reach of the health system.

Over the recent decade, the widespread availability of technology, including mobile phones, has had the potential to improve the efficiency and capacity of CHWs’ services delivery in resource limited settings.[5] Mobile-health (mHealth)–defined as the use of mobile and wireless technology for health–has the potential to improve health outcomes by addressing critical health systems constraints to service delivery, coverage and utilization.[6] Bangladesh, with an estimated 134 million mobile phone users, has declared its national vision for a Digital Bangladesh 2020 in 2007, which promotes the use of information technology (IT) for management, administration and governance to ensure transparency, accountability and accessibility at all levels of society and state.[7][8] Over the last decade, a wide array of mHealth initiatives have been piloted including collecting surveillance data, sending short message service (SMS) for health promotion, using sensors for diagnostic support or using calls for referrals or basic consultations. However, few have been scaled to the national level.[9]

In this vein, the mCARE I pilot program was initiated in 2011 and continued through August 2015 as a partnership between research, technical and implementation organizations, including Johns Hopkins University (JHU), mPower Social, and the JiVitA Project in Bangladesh.[10] The mCARE I pilot program was implemented in Gaibandha district of the Rangpur Division in northern Bangladesh, where the population is estimated 2.4 million with 80% residing in rural areas.[11] The aim of mCARE I is to develop and test a mobile phone-based system to improve communication and coordination between community health providers and the pregnant women they serve.

In this paper, we present findings on the incremental cost-effectiveness of two alternative mCARE strategies: (1) Comprehensive mCARE package program and (2) Basic mCARE package program. Both programs included a core package of census enumeration and pregnancy surveillance provided by an established cadre of digitally enabled community health research workers (CHRWs). In the comprehensive mCARE program, based on a woman’s last menstruation period, the system calculated gestational age and automatically scheduled four antenatal care (ANC) visits. The demand promotion interventions such as personally scheduled SMS reminders and home visit alerts were then additionally sent to all pregnant women and their household members as well as CHRWs from a server system to promote the pregnant women’s care-seeking of essential maternal and newborn health care services. In the basic mCARE program, on the other hand, there was no systematic demand promotion component other than a typical existing community-based service as a status quo. By exploring the incremental costs and consequences of these two alternative programs, study findings aim to contribute to overcoming the current paucity of data on the cost-effectiveness of digitally enabled CHW programs in low and middle-income countries (LMICs).

## Methods

### Setting

The mCARE I pilot program was implemented in Gaibandha district of the Rangpur Division in northern Bangladesh. Gaibandha has been part of the Johns Hopkins University JiVitA field site since 2001 and represents typical rural populations, agrarian characteristics and economic and public health indicators in Bangladesh. [12] Public health services are mainly provided through the following primary health care facilities: Satellite Clinics, Community Clinics, Family Welfare Clinics, and Upazila Health Complexes. Private sector services include informal and formal providers with some of the clinics supported by nongovernmental organizations.

### Study design

In a quasi-experimental design, the two comparable regions were selected in Gaibandha district for an intervention group and a comparison group. A cadre of 70 full-time, paid CHRWs– 35 CHRWs per group–were trained in the use of the mCARE mobile phone application, and they conducted a census of 11,836 all married women of reproductive age. Among them, 6,621 women were found to be eligible (i.e. excluding sterilized, menopausal, divorced or separated, husband died, refused, permanently moved or women died) for the study and consented to be visited every five weeks for pregnancy surveillance.

### Sample size

The sample size for the pregnant women was pre-determined to be at least more than 600 (300 per group) for another independent study on ANC utilization by the mCARE intervention (i.e. assuming 10–30% of ANC coverage increases by the intervention from 20% baseline coverage) based on a previous similar study.[13] From September 2013 to August 2014, a total of 695 pregnant women were enrolled and followed up over a year and 610 were eligible for the analysis with birth outcomes, 330 of those being part of a quasi-experimental intervention group and the remaining 280 part of a control group. Statistical power was calculated based on the two-sided test for proportions of adverse outcomes of two independent groups.

### Ethical approval

The mCARE I study received ethical approval from the Bangladesh Medical Research Council (Reference number: BMRC/NREC/2013-2016/375, dated 14/10/2015) and the Johns Hopkins Bloomberg School of Public Health Institutional Review Board (IRB00004522). For the data, subjects enrolled in the study completed written informed consent procedures from pregnant women.

### Program description

In both study groups, the following activities were performed in three phases: 1) Development phase (August 2011 to April 2013): partnership, program and system development; 2) Startup phase (May to August 2013): system optimization, training, community outreach and advocacy; and 3) Implementation phase (September 2013 to August 2015): census enumeration, pregnancy surveillance, SMS reminders, and home visit reminders. Specific activities are described in the Table A in S1 File.

During the implementation phase, the team sought to create a complete household enumeration by registering every resident with a unique identifier in the catchment area, and identifying married women of reproductive age (MWRAs, 15–45 years old), eligible for regular pregnancy surveillance in both intervention and comparison groups. Routine pregnancy surveillance was conducted every five weeks to identify pregnancies among MWRAs based on self-reported last menstrual period and urine pregnancy test confirmation.

In the intervention group (i.e. comprehensive mCARE package), pregnant women received two major intervention components–personally scheduled SMS reminders and CHRWs’ home visit reminders–implemented to promote care-seeking for ANC, skilled birth attendance or facility delivery, and postnatal care. First, based on a woman’s last menstruation period, the system calculated gestational age and automatically scheduled four ANC visits (+8–10, +12–27, +26–28 and +32–34 weeks) and sent SMS reminders on the scheduled dates to the pregnant woman (n = 330) and their household members who registered their mobile phone numbers (91%, 299 out of 330) as well as CHRWs (n = 35). Second, in addition to the scheduled SMS reminders, a CHRW mades a personal visit to the pregnant woman’s house to remind her of care-seeking, shortly in advance of the individually scheduled ANC visit dates. The second component ensured pregnant women receive at least a personal notice of ANC care seeking by CHRWs regardless of their ownership of or access to a mobile phone.

In the comparison group, these two intervention components were not implemented. All other activities, including partnership, system development, mobile phone procurement, training, community outreach, supervision, census enumeration, pregnancy surveillance, data processing and reporting throughout the development, startup and implementation phases, were conducted and shared in both study groups. This study design allowed for a systematic evaluation of the program outcomes–differing service coverage and health outcomes–based on a comparable denominator, which is the number of enrolled pregnant women, between the two groups.

### Costing

Economic costs were measured from a program perspective for the analytic time horizon of August 2011 to August 2015. Program costs were drawn from the financial records of the two implementing partners, mPower and JHU-JiVitA. Using standardized guidelines[14] and an ingredients-based approach, costs were divided into capital costs and recurrent costs and allocated into relevant program phases and activity categories (Table A-E in S1 File) through informant interviews with key program staff. All capital costs were annualized according to international or local estimates of each item’s life expectancy using a 3% annual discount rate.[15] Together with recurrent costs, these costs provided an estimate of the annual program costs of running a program or intervention. Office maintenance overhead costs were proportionally allocated to the activity costs in each stage based on staff workload ratios across the activity components. Total activity costs and unit cost per activity were calculated to determine the major drivers in each program phase and the difference between the intervention and control groups. Costs were presented in 2016 as the base year and in US dollars, adjusting for inflation according to consumer price indices from the IMF database.[16]

For any shared costs between the groups, we proportionally allocated the costs based on the size of relevant beneficiaries (i.e. pregnant women or CHRWs) in each group and standardized costs to a target population of 1 million per group.[17] Given a scenario of 1 million people in each group’s catchment area, we estimated the number of pregnant women as 15,000 in a given year, based on number of MWRA (560,000 in Gaibandha district in 2015)[11], fertility rate (66 per 1000 MWRAs) [18], abortion rate (29 per 1000 pregnant women)[19], and fetal loss rate (28 per 1000 births) [20] from national health statistics reports and an established calculation formula (see Table F and G in S1 File).[21] Based on the observed ratio of one CHRW to 10 pregnant women for annual pregnancy surveillance in the study site, 1,500 CHRWs were assumed to manage pregnancy surveillance and program intervention in a year. Accordingly, each unit cost was then extrapolated by the estimated number of CHRWs and pregnant women to calculate standardized costs per 1 million people.

### Effects

Health outcome was measured as numbers of deaths, including stillbirth, maternal, neonatal, and perinatal deaths which were drawn from the primary data collected from the household survey implemented during the mCARE I program from 2014 to 2015. We compared the respective adverse outcomes between the two groups and reported p-values based on chi-square tests. Similar to the cost analysis, we extrapolated the number of deaths to the estimated 15,000 pregnant women given a target population of 1 million per group. We calculated disability adjusted life years (DALYs) averted based on the number of deaths averted. Years of life lost (YLLs) were determined using the mean life expectancy of Bangladesh, which was 72 years. Due to the lack of morbidity data, years lived with disability (YLDs) were not included in the DALY calculation, assuming, based on literature, that their impact to total DALY measure is negligible.[22] The base case DALYs for newborns were discounted at a rate of annual 3% without age weighting.

### Cost-effectiveness analysis

Incremental cost-effectiveness ratios (ICERs) were examined based on comparisons of costs and effects between the study groups. To test the effect of simultaneous variations in multiple parameters, a Monte Carlo simulation was used to generate plausible values out of the assumed parameters distributions. A total of 1,000 iterations were generated using a Visual Basic macro in Excel. Cost-effectiveness acceptability curves were generated based on incremental willingness to pay to avert a death and compared to the GDP per capita of Bangladesh in 2016.

## Results

### Study participants characteristics

Table 1 illustrates general characteristics of the study population. Most basic biological, nutritional and economic statuses were similar in both groups, although a greater percentage of women in the comprehensive mCARE group were illiterate and from lower household wealth quintiles compared to those in the basic mCARE group.

**Table 1:** Characteristics of pregnant women (n = 610) by study group in the mCARE I program.

Characteristics of pregnant women		Comprehensive mCARE (n = 330)		Basic mCARE (n = 280)		P-value
		n	%	n	%	
Women’s age	<18 years	24	7%	25	9%	0.68
	18–35 years	294	89%	243	87%	
	>35 years	12	4%	12	4%	
Parity	Nulliparity	70	21%	71	25%	0.32
	1–2 births	187	57%	157	56%	
	>2 births	73	22%	51	18%	
Mid-upper arm circumference	<21.5 cm	42	13%	37	13%	0.86
	> = 21.5 cm	288	87%	243	87%	
Women’s literacy	Literate	189	57%	199	71%	<0.001
Household wealth index	Lowest quartile	90	27%	59	21%	0.01
	2^nd^ quartile	89	27%	60	21%	
	3^rd^ quartile	65	20%	84	30%	
	Highest quartile	81	25%	68	24%	

Data are number of pregnant women (n) and percentage (%). All p values relate to χ2 tests.

### Program costs

**Fig 1:**
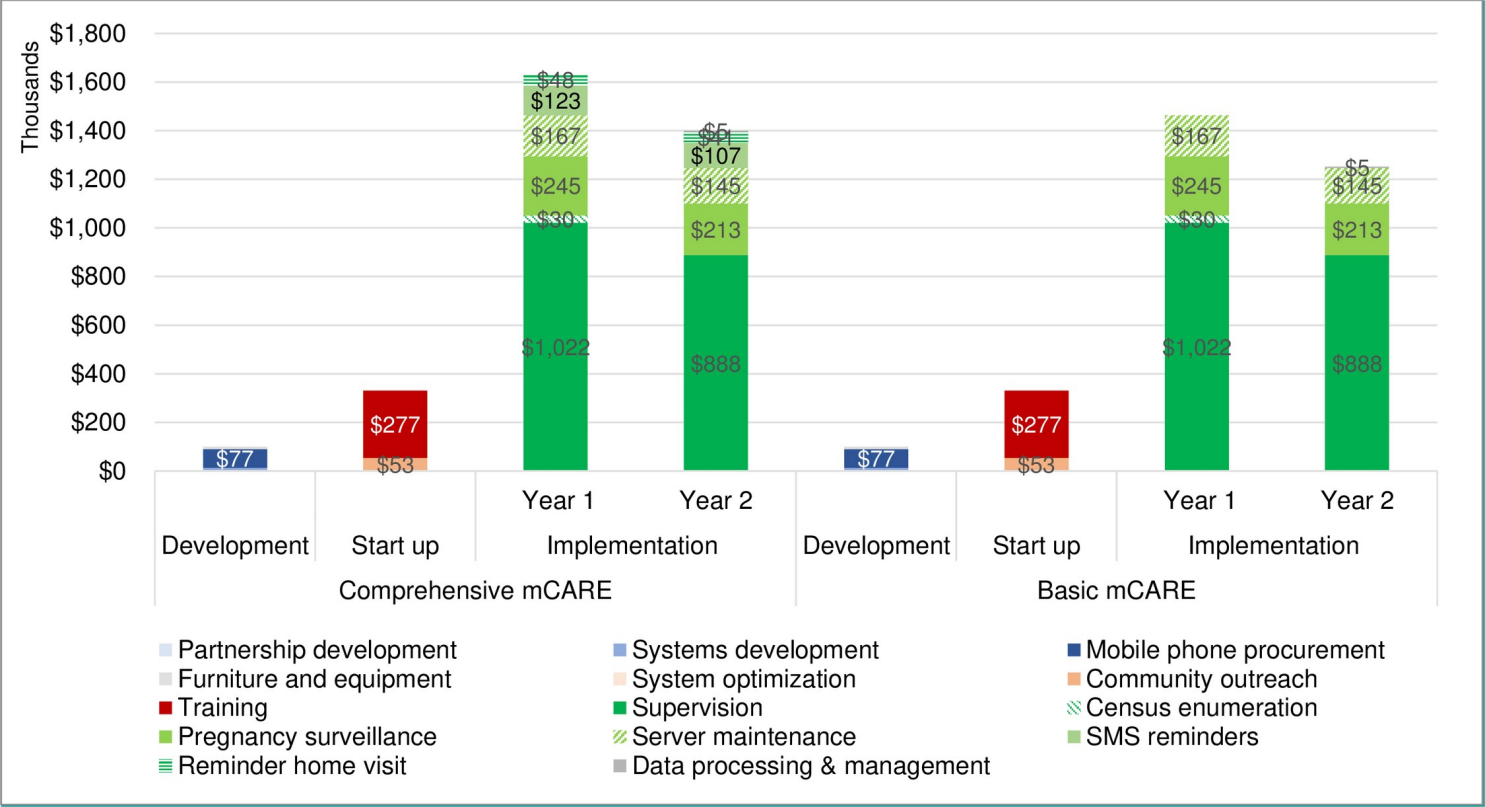
1 million population standardized program costs by study group in the mCARE I program.

Table 2 summarizes the program activity costs and unit costs. Fig 1 depicts standardized total program costs estimations in 1 million population by study group. In the development phase, mobile phone procurement and system development together make up 87% of the total development costs. In the start-up phase, training was the major cost as 84%. In the implementation phase, supervision was the major cost at 63% following mobile phone-based pregnancy surveillance at 15% and server maintenance at 10%. The intervention components, SMS reminders (8%) and home visits reminders (3%) together were estimated as only 11% of the total implementation costs. This is in part because of low unit costs such as an annual $7–8 per client for SMS reminders as SMS airtime cost is quite inexpensive in Bangladesh, and an annual $28–32 per CHRW for home visits as the local staff’s time costs for home visits were relatively small. Calculations of the annual cost for implementation including development and start-up with one year of implementation costs turned out to be $2.1 million for the comprehensive mCARE group. Overall, the total incremental cost of the comprehensive mCARE group compared to the basic mCARE group is estimated as $319,000 over the two years of implementation.

**Table 2:** Program activity costs by study group in the mCARE I program.

Program costs	mCARE I program		unit cost
	Comprehensive	Basic	(2016 USD$)
Number of populations	~20,000	~20,000	
Number of pregnant women (1 year)	330	280	
Number of CHRWs	35	35	
Development phase			
Partnership development	$3,000	$3,000	n/a
Systems development	$10,000	$10,000	n/a
Mobile phone procurement	$4,000	$4,000	$108/CHRW
Furniture and equipment	$10,000	$10,000	n/a
Start-up phase			
System optimization	$736	$736	n/a
Community outreach	$1,000	$1,000	$35/CHRW
Training	$6,000	$6,000	$184/CHRW
Implementation phase (1st year)			
Supervision	$24,000	$24,000	$681/CHRW
Census enumeration	$695	$695	$19/CHRW
Pregnancy surveillance	$6,000	$6,000	$163/CHRW
Server maintenance	$4,000	$4,000	$111/CHRW
SMS reminders	$1,000	$0	$8/Client
Reminder home visit	$1,000	$0	$32/CHRW
Implementation phase (2nd year)			
Supervision	$21,000	$21,000	$592/CHRW
Pregnancy surveillance	$5,000	$5,000	$142/CHRW
Server maintenance	$3,000	$3,000	$97/CHRW
SMS reminders	$1,000	$0	$7/Client
Reminder home visit	$966	$0	$28/CHRW
Data processing & management	$10,000	$10,000	n/a
**Total program cost**^**1**^	**$114,000**	**$110,000**	

1. Costs are rounded up to the thousand unit. See Table F in S1 File for the full cost information.

### Effects

Greater adverse pregnancy outcomes were observed in basic mCARE group compared to comprehensive mCARE group, except for the stillbirths, while it was not statistically significant. While both neonatal deaths and miscarriages were determined as statistically greater in the control group compared to the intervention group, we did not include miscarriages into the cost effectiveness analysis as the mCARE care seeking reminder intervention (four times throughout the pregnancy stages) may not fully affect this outcome (Table 3). Once adjusting for a population of 1 million, we estimated a difference of 354 (uncertainty range 145–571) newborn deaths averted between the intervention and comparison groups.

**Table 3:** Adverse pregnancy outcomes and 1 million population standardization.

Adverse outcomes	mCARE I program		P-value	1 Million population standardized estimation	
	Comprehensive (pregnant women = 330)	Basic (pregnant women = 280)		Comprehensive (pregnant women = 15,000)	Basic (pregnant women = 15,000)
Maternal death	0 (0.00%)	1 (0.36%)	0.28	0	54
Stillbirth	13 (3.99%)	7 (2.50%)	0.32	591	375
Newborn death	4 (1.21%)	10 (3.57%)	0.04	182	536
Miscarriage	5 (1.50%)	14 (5.00%)	0.01	227	750

Data are number of deaths (n) and percentage (%). All p values relate to χ2 tests.

### Incremental cost effectiveness analyses

**Fig 2:**
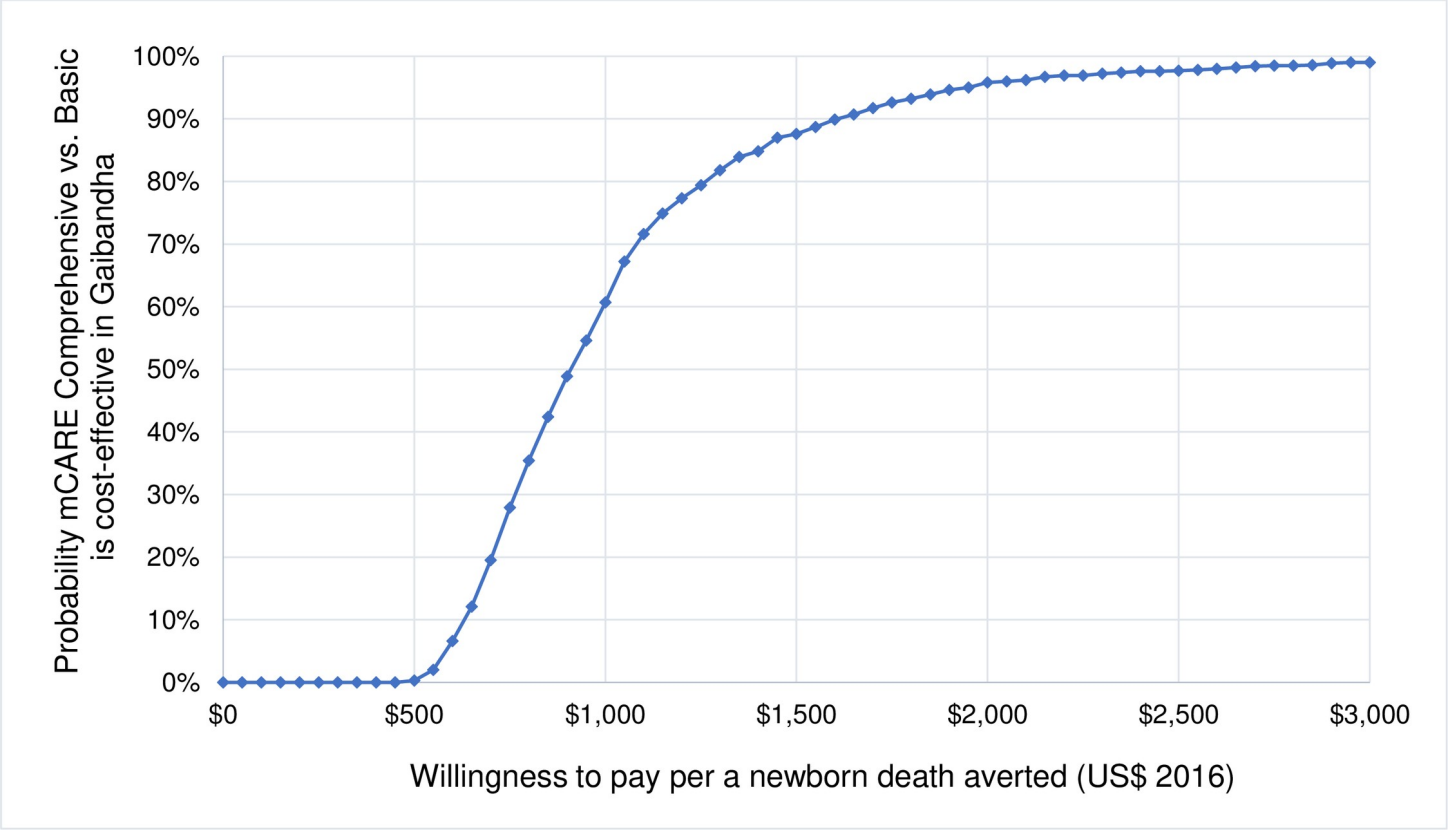
Cost effectiveness acceptability curve showing 88% probability of being cost effective of mCARE comprehensive program compared to basic program at a threshold value defined as Bangladesh GDP per capita ($1,500).

Our results indicate that the comprehensive mCARE group (with SMS and home visit reminders) was highly cost-effective compared to the basic mCARE group–with $901 per death averted and $31 per DALY averted (Table 4). The cost-effectiveness acceptability curve quantifies the probability that the program would be highly cost effective as 88% at a threshold value of $1,500, the Bangladesh GDP per capita in 2016 (Fig 2).

**Table 4:** Summary of incremental cost effectiveness ratios between mCARE comprehensive and basic programs (2011–2015) based on 1 million population standardized estimations.

Primary Health and Economic Outcomes (95% Uncertainty ranges)					
Programs	Neonatal deaths averted	DALY averted	Total program cost in thousand USD (2 years)	Cost per newborn death averted	Cost per DALY averted
Comprehensive mCARE	182 (112~251)	5,369 (3,372–7,499)	$3,465 ($3,240 -$3,693)		
Basic mCARE	536 (332~740)	15,812 (9,630–21,733)	$3,146 ($2,922-$3,373)		
Incremental (Comprehensive-Basic)	354 (145–571)	10,443 (3,931–16,850)	$319 ($290~$348)	$901 ($557~$2,378)	$31 ($19-$81)

## Discussion

Despite a relatively small difference in mortality impact between intervention and control groups, study findings suggest that the comprehensive mCARE package may be highly cost-effective at $31 per DALY averted. Key drivers of program costs included supervision and training, which were associated with increasing numbers of CHRWs and efforts to adapt to new practices and protocols. The results suggest that once surveillance is initially conducted using mobile phone, marginal costs of adding personally scheduled SMS and home visit reminders to promote care-seeking are almost negligible, but this small investment can make a life-saving impact in low-resource settings.

Based on the study design, it is important to clarify that our work demonstrates incremental benefits of adding SMS and home visit reminders, rather than an entire mHealth package program. Since our comparison group, the basic mCARE package, also used mobile phone for pregnancy surveillance, our finding does not present mCARE benefits compared to current paper-based practices. However, as stated above, this allows for a systematic comparison and evaluation of the mHealth intervention (in particular the SMS and home visit reminders)’s impact on service coverage improvement and mortality reduction based on compatible population denominators between the intervention and control groups. Further, we took a program perspective of implementing an mHealth intervention, and thus did not include household costs or service provision costs associated with the intervention. However, in this setting where ANC services are largely free of charge in all public facilities and only take minimal costs (e.g. $0.06 per ANC) in some NGO clinics, we consider that user or provider costs would not affect our conclusions on cost effectiveness.

While there is growing evidence of mHealth impact on maternal, newborn and child health (MNCH) in LMICs, there is yet little systematic and rigorous mHealth economic evaluation research being done in LMICs.[23] Most existing economic evaluation studies include mHeath strategies of SMS reminders for treatment adherence for HIV/ART treatment[24], malaria[25, 26] and TB treatments[27] and smoking cessation.[28] Some studies used mHealth strategies for data collection[29, 30] family planning training[31] and telephone support for breastfeeding [32] with some processing time and quality improvement indicators. However, most studies did not address uncertainty for their study findings and no study has yet examined value for money regarding SMS reminders on top of a population-based digital surveillance system. This analysis is thus an important contribution to the field, as it evaluates major mHealth strategies for MNCH interventions, which are surveillance data collection by frontline health workers and SMS reminders to clients. As a similar example, the study by Lund and colleagues [33] added a voucher system to an mHealth program to improve ANC care-seeking and showed statistically significant coverage uptake and perinatal mortality reduction in Tanzania. As service delivery strategies, our finding suggests mHealth strategies can be complementary to or may enhance the cost effectiveness of community-based maternal and newborn care interventions as demand promotion strategies.

### Limitations

Our study has some limitations. First, the mCARE I study was a pilot study on a quasi-experimental design, which did not provide enough statistical power and adjustment for confounding factors in evaluating mortality impact. To detect the observed difference in newborn mortality (i.e.1.21% in the control group and 3.57% in the intervention group), we had a power of 46%. Thus, the findings may be suggestive and not definitive. Yet, considering a greater proportion of pregnant women who are in lower wealth quintiles and illiterate in the intervention group than in the control group in this quasi experimental setting, the outcome may have been conservative estimates. Given the fact that the mHealth intervention was a reminder for care seeking, not provision of care itself, the health impact can be influenced by access to and quality of the local health facilities and the pregnant women’s care-seeking characteristics. The enabling conditions of a health system, such as level of mobile phone penetration and ownership in the community, stable electricity and network connection, and an available community health workforce to manage operations at scale are also critical aspects to consider.

Second, our cost adjustment for standardized estimations to a population of 1 million may not systematically incorporate potential changes with scaling up. The proportional extrapolation based on the relevant numbers of user and beneficiaries may not consider potential productivity or efficiency gains or additional system requirements associated with mHealth programs at scale over time. Besides the mortality impact, mHealth is expected to provide substantial benefits in operational practices with improved communications, increased workers’ empowerment, and enhanced accuracy, quality, and efficiency in process indicators, shown in many qualitative studies.[34] However, the currently limited amount of evidence makes it challenging for systematic quantification of these features for cost-effectiveness analyses. Considering these direct benefits as well as positive externalities, our measure of cost per death averted may be considered a conservative estimate of the value of the mHealth strategy.

## Conclusions

The study contributes to the currently available economic evaluation data on mHealth interventions in Bangladesh and globally. Study findings suggest that the addition of SMS and home visit reminders based on a mobile phone-facilitated pregnancy surveillance system may be cost-effective. Future research may consider setting a comparison group as the current status quo (e.g. paper-based practice) on the ground to examine comparative costs and consequences of implementing the mHealth package as a whole. We suggest that incorporating simple mHealth strategies such as SMS and home visit reminders to proven community-based delivery strategies may improve service utilization and program cost effectiveness in low-resource settings.

## Supporting information

S1 File (DOCX)
