# Supplementary material for: Costs and cost-effectiveness analyses of mCARE strategies for promoting care seeking of maternal and newborn health services in rural Bangladesh
Source: PLoS One. 2019 Oct 1;14(10):e0223004. doi: 10.1371/journal.pone.0223004 (PMC6773420; doi:10.1371/journal.pone.0223004)
Supplement: S1 File — (DOCX) [file pone.0223004.s001.docx]

**Supporting Information (S1 File)**

**S1 Figure A. Analytic approach of the cost effectiveness analyses**

| **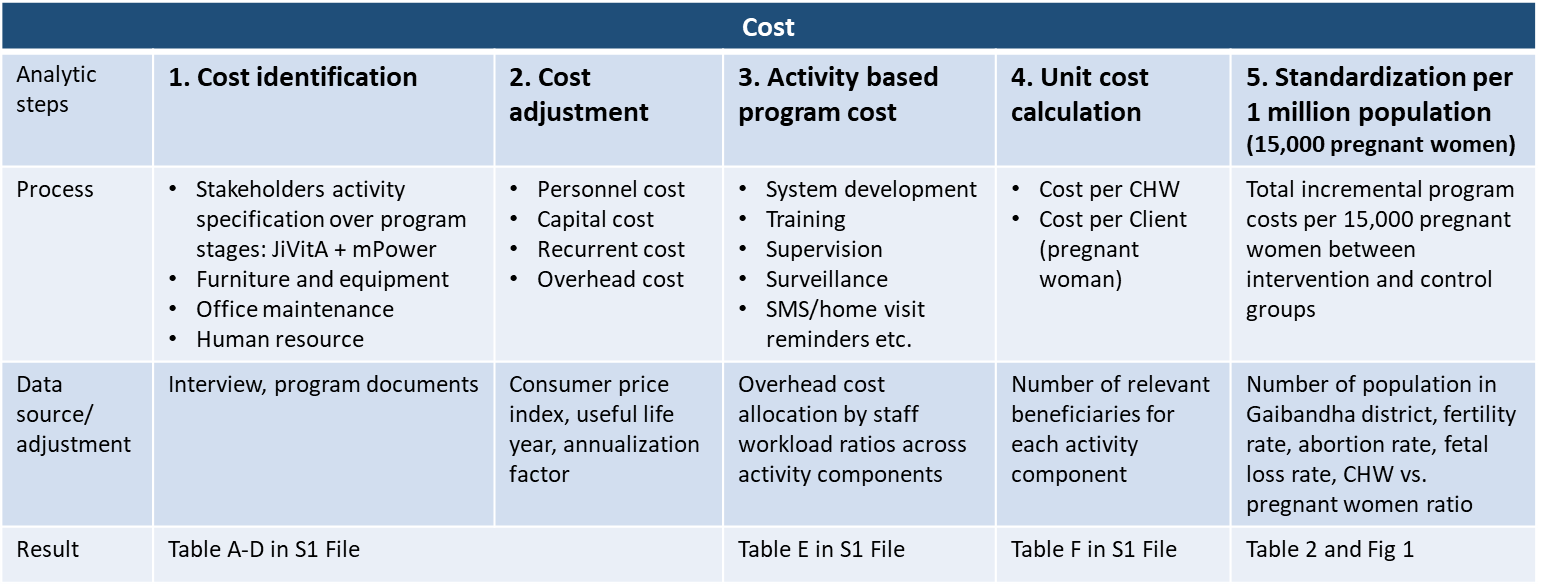**  **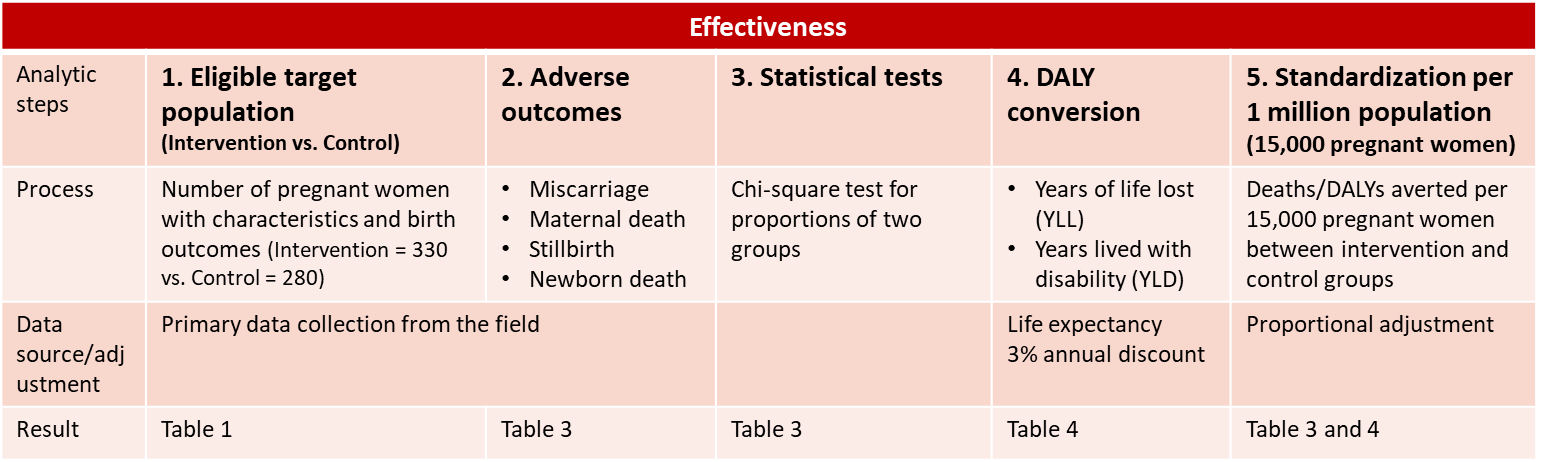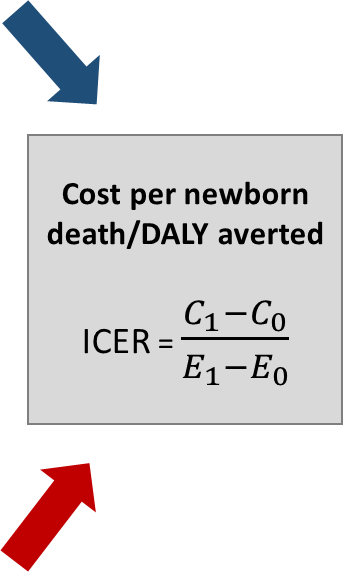** |
| --- |

S2 Table A. Activity based program costs definitions over mCARE I program development, start up, and implementation for comprehensive mCARE program (C) and basic mCARE program (B)

| **Program activities** | **Activity descriptions** | **Study arms** | | **Cost type** |
| --- | --- | --- | --- | --- |
|  |  | C | B |  |
| **Development (August 2011-April 2013): 21 months** | |  |  |  |
| Partnership development | JHU/ JiVitA / mPpwer held leadership meetings and an official launch on mCARE project among central, regional, and district health management teams. JHU contracted mPower as technical system developer. | √ | √ | Capital costs  (3 years of useful time; annualized) |
| Systems development | mPower prepared systems requirement specifications; developed scheduling logic, skip patterns, question type feedback; development of detailed technical specifications (end user centered design criteria; field health workers’ workflow and information flow analysis) | √ | √ |  |
| Mobile phone procurement | Mobile phones procurement (n=70) and distribution; system embedment | √ | √ |  |
| Furniture and equipment | JiVitA/mPower computers, desks, chairs, cabinets, vehicles etc. | √ | √ |  |
| Office maintenance | JiVitA/mPower office rent, office supplies, utilities (electricity, gas, water etc.) | √ | √ |  |
| **Start up (May-August 2013): 4 months** | |  |  |  |
| System optimization | mPower developed system prototype and testing and outsourced SMS service component | √ | √ | Capital costs  (3 years of useful time; annualized) |
| Training | JiVitA / mPower prepared training manuals; conducted 3 week trainings with mobile phones to 70 CHWs; evaluated CHWs’ performance and acceptability; printed out survey forms; prepared data management/ data entry screen generation; field testing | √ | √ |  |
| Community outreach | JHU/JiVitA established an MOU with the district government (Directorate General of Family Planning); held an official launching meeting by inviting local leaders and partners in Gaibandha; distributed mCARE brochure to community | √ | √ |  |
| Office maintenance | JiVitA/mPower office rent, office supplies, utilities (electricity, gas, water etc.) | √ | √ |  |
| **Implementation (September 2013-August 2014): 12 months** | |  |  |  |
| Supervision | JiVitA senior management team and field supervisor monitored field implementation activities and progress through weekly meetings, field visiting, data monitoring, quality control activities etc. | √ | √ | Recurrent costs |
| Census enumeration | Field distributers visited all households for census enumeration, married women of reproductive age (MWRA) registration through mobile phones for 5 weeks. (i.e. 1 field distributor covers on average 300-350 households identifying 100-120 MWRAs in 5 weeks.) | √ | √ |  |
| Pregnancy surveillance | Field distributors visited eligible couple's households for pregnancy registration, to receive consent for the study through mobile phones for 10 months. (i.e. 1 field distributor identifies about 8-10 pregnant women in a year) | **√** | **√** |  |
| SMS reminders | SMS automatically sent from server to pregnant women's phones at their expected ANC 1-4 dates. | **√** |  |  |
| Home visit reminders | Field distributers visit pregnant women's houses four days before their scheduled ANC 1-4 dates to remind them and to encourage ANC care-seeking. | **√** |  |  |
| Server maintenance | Server automatically sent scheduled SMS to pregnant women and update workflow (e.g. list of households to be visited in the week) to CHWs | **√** | **√** |  |
| Office maintenance | JiVitA/mPower office rent, office supplies, utilities (electricity, gas, water etc.) | **√** | **√** |  |
| **Implementation (September 2014-August 2015): 12 months** | |  |  |  |
| Supervision | JiVitA senior management team and field supervisor monitored field implementation activities and progress through weekly meetings, field visiting, data monitoring, quality control activities etc. | **√** | **√** | Recurrent costs |
| Pregnancy surveillance | Field distributors visited all households for pregnancy registration and to obtain consent for the study through mobile phones for 10 months. | **√** | **√** |  |
| SMS reminders | SMS automatically sent from server to pregnant women's phones at their expected ANC 1-4 dates. | **√** |  |  |
| Home visit reminders | Field distributers visited pregnant women's houses four days before their ANC 1-4 dates to remind them and to encourage ANC care-seeking. | **√** |  |  |
| Server maintenance | Server to automatically send scheduled SMS to pregnant women and update workflow (e.g. list of households to be visited in the week) to field distributers | **√** | **√** |  |
| Data processing & management | mPower provided technical assistance, trouble shooting, data cleaning etc; JiVitA conducted data cleaning, analysis and reporting | **√** | **√** |  |
| Office maintenance | JiVitA/mPower office rent, office supplies, utilities (electricity, gas, water etc.) | √ | √ |  |

To consider the broader health systems implications of adopting a mobile health strategy in Bangladesh, we conducted a detailed activity-based costing to identify who is involved, what the new resource requirements are, how and why the processes are changed, and to what extent these changes are occurring over the full course of program development, preparation and implementation. With this approach, we conceptualized and categorized major activity components based on their purpose and characteristics and identified the costs of the activity item based on relevant staff salaries, working months, and their levels of effort.

In this adjustment, we carefully reviewed characteristics of each cost item such as activity-based costs and overhead costs and divided the costs into capital and variable costs. Capital costs, unlikely to change based on the number of beneficiaries, include partnership development, system development, system optimization, data processing and analyses, furniture and equipment procurement, and office maintenance during development and start-up phases. Variable costs include mobile phone procurement, training, community outreach, supervision, server maintenance, census enumeration, pregnancy surveillance, SMS reminders, and home visit reminders. In terms of the variable costs, unit costs were calculated per CHRW, as the unit because most program activity costs are directly drawn from their work capacity, time, and salary. The SMS air-time costs, however, are calculated per client (i.e. pregnant woman), as shown in the manuscript Table 2.

**S3 Table B. Furniture and equipment cost**

| Furnitures and Equipment | Cost per item (BDT) | Consumer Price Index adjusted | Number of staff / quantities | Total costs (BDT) | Total costs (USD) | Life expectancy (years) | Annualization factor | Annualized Cost (2015, USD) |
| --- | --- | --- | --- | --- | --- | --- | --- | --- |
| **JiVitA** | | | | | | | | |
| Desks | 6,500 | 8,495 | 26 | 220,880 | 2,871 | 10 | 8.53 | 337 |
| Chairs | 3,500 | 4,574 | 26 | 118,935 | 1,546 | 10 | 8.53 | 181 |
| Cabinets | 12,000 | 15,684 | 17 | 266,624 | 3,466 | 10 | 8.53 | 406 |
| Computer | 45,000 | 58,814 | 12 | 705,770 | 9,175 | 5 | 4.58 | 2,003 |
| Laptop | 50,000 | 65,349 | 5 | 326,746 | 4,248 | 5 | 4.58 | 927 |
| Motorbike | 142,000 | 185,591 | 2 | 371,183 | 4,825 | 10 | 8.53 | 566 |
| Table 1 | 6,500 | 8,495 | 5 | 42,477 | 552 | 10 | 8.53 | 65 |
| Table 2 | 8,000 | 10,456 | 12 | 125,470 | 1,631 | 10 | 8.53 | 191 |
| Chair 1 | 3,500 | 4,574 | 5 | 22,872 | 297 | 10 | 8.53 | 35 |
| Chair 2 | 1,500 | 1,960 | 25 | 49,012 | 637 | 10 | 8.53 | 75 |
| Bench | 4,000 | 5,228 | 10 | 52,279 | 680 | 10 | 8.53 | 80 |
| Cabinets | 13,000 | 16,991 | 5 | 84,954 | 1,104 | 10 | 8.53 | 129 |
| Computer | 45,000 | 58,814 | 1 | 58,814 | 765 | 5 | 4.58 | 167 |
| Motobike | 142,000 | 185,591 | 5 | 927,957 | 12,063 | 10 | 8.53 | 1,414 |
| Bicycle | 6,000 | 7,842 | 25 | 196,047 | 2,549 | 10 | 8.53 | 299 |
| Rack | 3,000 | 3,921 | 17 | 66,656 | 867 | 10 | 8.53 | 102 |
| **Total furniture and equipment cost** |  |  |  | 57,456 |  |  |  | **6,977** |
| **mPower** | | | | | | | | |
| Chair | 6,000 | 7,842 | 15 | 90,000 | 1,170 | 10 | 8.53 | 137.16 |
| Desk | 7,000 | 9,149 | 15 | 105,000 | 1,365 | 10 | 8.53 | 160.02 |
| Computer/laptop | 55,000 | 71,884 | 15 | 825,000 | 10,725 | 5 | 4.58 | 2341.70 |
| **Total furniture and equipment cost** |  |  |  |  |  |  |  | **2,639** |

**S4 Table C. Office maintenance costs**

| Office maintenance costs | Average monthly maintenance cost (BDT) | Year | 2011 | 2012 | 2013 | 2014 | 2015 | Total costs | | Annualization factor^1^ | Annualized Cost (2016, USD) |
| --- | --- | --- | --- | --- | --- | --- | --- | --- | --- | --- | --- |
|  |  | Months | 5 | 12 | 12 | 12 | 6 | BDT | USD |  |  |
| JiVitA |  | LOE | 0% | 15% | 20% | 30% | 10% |  |  |  |  |
| Office rent, | 268,141 |  | 0 | 482,653 | 643,537 | 965,306 | 160,884 | 2,252,380 | 29,281 |  |  |
| Electricity bills, gas, purchases, printing | 50,095 |  | 0 | 90,171 | 120,227 | 180,341 | 30,057 | 420,796 | 5,470 |  |  |
| Wage | 46,188 |  | 0 | 83,138 | 110,851 | 166,277 | 27,713 | 387,979 | 5,044 |  |  |
| Fuel, | 128,228 |  | 0 | 230,811 | 307,748 | 461,622 | 76,937 | 1,077,117 | 14,003 |  |  |
| Travel and per diem, | 118,943 |  | 0 | 214,098 | 285,464 | 428,196 | 71,366 | 999,123 | 12,989 |  |  |
| Repair and services, | 44,973 |  | 0 | 80,951 | 107,935 | 161,903 | 26,984 | 377,773 | 4,911 |  |  |
| Bank charges, | 4,059 |  | 0 | 7,306 | 9,741 | 14,612 | 2,435 | 34,094 | 443 |  |  |
| Supplies and other services, | 235,493 |  | 0 | 423,887 | 565,182 | 847,773 | 141,296 | 1,978,137 | 25,716 |  |  |
| Stationaries. | 53,873 |  | 0 | 96,971 | 129,295 | 193,942 | 32,324 | 452,531 | 5,883 |  |  |
| Program phases |  |  |  |  |  |  |  |  |  |  |  |
| Development (Aug 2011- April 2013) |  |  |  | 1,709,985 | 759,993 |  |  | 2,469,979 | 32,110 | 2.83 | 11,346 |
| Start up (May 2013-Aug 2013) |  |  |  |  | 759,993 |  |  | 759,993 | 9,880 | 2.83 | 3,491 |
| Implementation (Sep 2013-Aug 2015) |  |  |  |  | 759,993 | 3,419,970 | 569,995 | 4,749,959 | 61,749 | 2.83 | 21,820 |
| mPower | | LOE | 30% | 40% | 40% | 10% | 5% | BDT | USD |  |  |
| House Rent | 300,000 |  | 450,000 | 1,440,000 | 1,440,000 | 360,000 | 90,000 | 3,780,000 | 49,140 |  |  |
| Maintenance Charges | 46,500 |  | 69,750 | 223,200 | 223,200 | 55,800 | 13,950 | 585,900 | 7,617 |  |  |
| Internet Bandwidth | 26,000 |  | 39,000 | 124,800 | 124,800 | 31,200 | 7,800 | 327,600 | 4,259 |  |  |
| Photocopies; office supplies; stationery/printer toner etc. | 10,000 |  | 15,000 | 48,000 | 48,000 | 12,000 | 3,000 | 126,000 | 1,638 |  |  |
| Utilities (Water, Electricity, Gas bill etc.) | 61,350 |  | 92025 | 294480 | 294480 | 73620 | 18405 | 773010 | 10,049 |  |  |
| Telecommunication (Telephone Bill) | 1,500 |  | 2250 | 7200 | 7200 | 1800 | 450 | 18900 | 246 |  |  |
| Support Staff | 40,000 |  | 60000 | 192000 | 192000 | 48000 | 12000 | 504000 | 6,552 |  |  |
| Postage & Courier, Bank Charges | 2,500 |  | 3750 | 12000 | 12000 | 3000 | 750 | 31500 | 410 |  |  |
| Program phases |  |  |  |  |  |  |  |  |  |  |  |
| Development (Aug 2011- April 2013) |  |  | 731,775 | 2,341,680 | 780,560 |  |  | 3,854,015 | 50,102 | 2.83 | 17,704 |
| Start up (May 2013-Aug 2013) |  |  |  |  | 780,560 |  |  | 780,560 | 10,147 | 2.83 | 3,586 |
| Implementation (Sep 2013-Aug 2015) |  |  |  |  | 780,560 | 585,420 | 146,355 | 1,512,335 | 19,660 | 2.83 | 6,947 |

1. Annualization factor (2.83) is based on 3 years of program start up and implementation period.

**S5 Table D. Personnel costs**

|  | Activity specification | Personnel cost^1^ | Consumer Price Index adjusted | Total costs (2016, BDT) | Total costs (2016, USD) | Life expectancy (years) | Annualization factor | Annualized program cost (2016, USD) |
| --- | --- | --- | --- | --- | --- | --- | --- | --- |
| Capital cost | JiVitA |  |  |  |  |  |  |  |
|  | Partnership & consensus building | 289,575 | 470,559 | 470,559 | 6,117 | 3 | 2.83 | 2,162 |
|  | Systems development | 943,463 | 1,533,127 | 1,533,127 | 19,931 | 3 | 2.83 | 7,043 |
|  | Community campaign & awareness | 192,267 | 312,434 | 312,434 | 4,062 | 3 | 2.83 | 1,435 |
|  | Training | 1,003,178 | 1,630,165 | 1,630,165 | 21,192 | 3 | 2.83 | 7,488 |
|  | Mobile phone procurement^2^ | N/A | N/A | 783,052 | 10,180 | 3 | 2.83 | 3,597 |
|  | mPower |  |  |  |  |  |  |  |
|  | Partnership & consensus building | 142,244 | 181,362 | 181,362 | 2,358 | 3 | 2.83 | 833 |
|  | Systems requirement specifications | 372,969 | 475,538 | 475,538 | 6,182 | 3 | 2.83 | 2,184 |
|  | Prototype development | 121,043 | 154,331 | 154,331 | 2,006 | 3 | 2.83 | 709 |
|  | Dashboard design & development | 121,267 | 154,616 | 154,616 | 2,010 | 3 | 2.83 | 710 |
|  | System testing and optimization | 145,848 | 185,958 | 185,958 | 2,417 | 3 | 2.83 | 854 |
| Recurrent cost | JiVitA |  |  |  |  |  |  |  |
|  | Supervision | 3,643,105 | 4,761,538 | 4,761,538 | 59,797 | 3 | 2.83 | 21,130 |
|  | Census enumeration | 36,254 | 47,385 | 134,000 | 1742 | 3 | 2.83 | 616 |
|  | Pregnancy surveillance | 298,334 | 389,923 | 1,103,488 | 14,345 | 3 | 2.83 | 5,069 |
|  | SMS reminder^3^ | N/A | 130,400 | 130,400 | 1,695 | 3 | 2.83 | 599 |
|  | Reminder home visit | 46,356 | 49,462 | 107,200 | 1,394 | 3 | 2.83 | 643 |
|  | Data processing & analyses | N/A | N/A | N/A | 24,000 | 3 | 2.83 | 8,481 |
|  | mPower |  |  |  |  |  |  |  |
|  | Server maintenance^4^ | N/A | 750,000 | 750,000 | 9,750 | 3 | 2.83 | 3,445 |
|  | Data processing & management | 253,403 | 253,403 | 253,403 | 3,294 | 3 | 2.83 | 1,164 |

1. Personnel costs were translated into relevant activity costs based on number of staff, respective monthly salaries, working months, and level of effort (%) to the specific tasks in the mCARE project.
2. Mobile phone procurement costs include mobile phone cost ($86 per mobile phone for 70 CHRWs) and other procurement fees.
3. SMS reminders cost include unit cost per SMS message ($0.06) for 4 ANC/child delivery/3 PNC reminders per client (including pregnant women and CHRWs), monthly connecting charge ($5 per month), and total number of months.
4. Server maintenance cost include server hosting fee ($105 per month) and server equipment purchase and maintenance cost ($325 per month).

**S6 Table E. Activity based program costs (2016 USD$)**

| Cost types | Cost categories | JiVitA | mPower | JiVitA + mPower | Overhead cost allocation^1^ | Program costs (annualized) |
| --- | --- | --- | --- | --- | --- | --- |
| Personnel cost | Partnership development | 2,162 | 833 | 2,995 | 11% | $6,322 |
| Personnel cost | Systems development | 7,043 | 2,893^2^ | 9,936 | 38% | $20,976 |
| Capital cost | Mobile phone procurement | 3,597 | N/A | 3,597 | 14% | $7,594 |
| Capital cost | Furniture and equipment | 6,977 | 2,639 | 9,616 | 37% | $20,300 |
| Overhead cost | Office maintenance | 11,346 | 17,704 | 29,050 |  |  |
| Aug, 2011-April, 2013 | Total development costs (A) |  |  |  |  | $55,194 |
| Personnel cost | System optimization | N/A | 854 | 854 | 9% | $1,472 |
| Personnel cost | Community outreach | 1,435 | N/A | 1,435 | 15% | $2,474 |
| Personnel cost | Training | 7,488 | N/A | 7,488 | 77% | $12,908 |
| Overhead cost | Office maintenance | 3,491 | 3,586 | 7,077 |  |  |
| May, 2013-Aug, 2013 | Total start-up costs (B) |  |  |  |  | $16,855 |
| Personnel cost | Supervision | 21130 | N/A | 21,130 | 65% | $47,684 |
| Personnel cost | Census enumeration | 616 | N/A | 616 | 2% | $1,389 |
| Personnel cost | Pregnancy surveillance | 5,069 | N/A | 5,069 | 16% | $11,440 |
| Recurrent cost | Server maintenance | N/A | 3445 | 3,445 | 11% | $7,775 |
| Recurrent cost | SMS reminders | 599 | N/A | 599 | 4% | $1,352 |
| Personnel cost | Reminder home visit | 492 | N/A | 492 | 3% | $1,111 |
| Overhead cost | Office maintenance | 21,820 | 6,947 | 28,767 |  |  |
| Sep, 2013-Aug, 2014 | Total implementation costs (C) |  |  |  |  | $70,752 |
| Personnel cost | Supervision | 21,130 | N/A | 21,130 | 50% | $41,460 |
| Personnel cost | Pregnancy surveillance | 5,069 | N/A | 5,069 | 12% | $9,946 |
| Recurrent cost | Server maintenance | N/A | 3,445 | 3,445 | 8% | $6,760 |
| Recurrent cost | SMS reminders | 599 | N/A | 599 | 3% | $1,175 |
| Personnel cost | Reminder home visit | 643 | N/A | 492 | 2% | $966 |
| Personnel cost | Data processing & management | 8,481 | 1,164 | 10,550 | 25% | $20,700 |
| Overhead cost | Office maintenance | 21,820 | 6,947 | 28,767 |  |  |
|  |  |  |  |  |  | $81,008 |

1. Overhead cost was allocated to the key activity costs based on average proportional personnel cost (%) on each activity group component.
2. System development costs in mPower include costs of systems requirement specifications, prototype development, and dashboard design and development

S7 Table F. 1 million population standardized program costs by study group in the mCARE I program

| Program coverage/costs | Program costs (Annualized) | mCARE I program | | unit cost/unit  (2016 USD$) | 1 Million standardized estimation | |
| --- | --- | --- | --- | --- | --- | --- |
|  |  | Comprehensive | Basic |  | Comprehensive | Basic |
| Number of populations | ~40000 | ~20,000 | ~20,000 |  | 1 million | 1 million |
| Number of pregnant women (1 year) | 610 | 330 | 280 |  | 15,000 | 15,000 |
| Number of CHRWs | 70 | 35 | 35 |  | 1500 | 1500 |
| Development phase | | | | | | |
| Partnership development | $6,322 | $3,161 | $3,161 | n/a | $3,161 | $3,161 |
| Systems development | $20,976 | $10,488 | $10,488 | n/a | $10,488 | $10,488 |
| Mobile phone procurement | $7,594 | $3,797 | $3,797 | $108/CHRW | $77,080 | $77,080 |
| Furniture and equipment | $20,300 | $10,150 | $10,150 | n/a | $10,150 | $10,150 |
| **Total development costs (A)** | **$55,192** | **$27,596** | **$27,596** |  | **$100,879** | **$100,879** |
| Start-up phase | | | | | | |
| System optimization | $1,472 | $736 | $736 | n/a | $736 | $736 |
| Community outreach | $2,474 | $1,237 | $1,237 | $35/CHRW | $53,013 | $53,013 |
| Training | $12,908 | $6,454 | $6,454 | $184/CHRW | $276,603 | $276,603 |
| **Total start-up costs (B)** | **$16,854** | **$8,427** | **$8,427** |  | **$330,352** | **$330,352** |
| Implementation phase (1st year) | | | | | | |
| Supervision | $47,684 | $23,842 | $23,842 | $681/CHRW | $1,021,816 | $1,021,816 |
| Census enumeration | $1,390 | $695 | $695 | $19/CHRW | $29,767 | $29,767 |
| Pregnancy surveillance | $11,440 | $5,720 | $5,720 | $163/CHRW | $245,133 | $245,133 |
| Server maintenance | $7,776 | $3,888 | $3,888 | $111/CHRW | $166,608 | $166,608 |
| SMS reminders | $1,352 | $1,352 | $0 | $8/Client | $122,893 | $0 |
| Reminder home visit | $1,111 | $1,111 | $0 | $32/CHRW | $47,628 | $0 |
| Total implementation 1^st^ year costs (C) | $70,753 | $36,608 | $34,145 |  | $1,633,845 | $1,463,324 |
| **Total program 1^st^ year costs (A+B+C)** | **144,171** | **72,631** | **71,540** |  | **$2,065,076** | **$1,894,555** |
| Implementation phase (2nd year) | | | | | | |
| Supervision | $41,460 | $20,730 | $20,730 | $592/CHRW | $888,414 | $888,414 |
| Pregnancy surveillance | $9,946 | $4,973 | $4,973 | $142/CHRW | $213,130 | $213,130 |
| Server maintenance | $6,760 | $3,380 | $3,380 | $97/CHRW | $144,857 | $144,857 |
| SMS reminders | $1,175 | $1,175 | $0 | $7/ Client | $106,849 | $0 |
| Reminder home visit | $966 | $966 | $0 | $28/CHRW | $41,410 | $0 |
| Data processing & management | $20,700 | $10,350 | $10,350 | n/a | $5,275 | $5,275 |
| **Total Implementation 2^nd^ year costs (D)^1^** | **$81,007** | **$41,574** | **$39,433** | **n/a** | **$1,399,935** | **$1,251,676** |
| **Total program 2^nd^ year costs (A+B+D)** | **$153,053** | **$77,597** | **$75,456** |  | **$1,831,166** | **$1,682,907** |
| **Total program cost over 2 years (A+B+C+D)** | **$223,806** | **$114,205** | **$109,601** |  | **$3,465,011** | **$3,146,231** |

1. Some activity costs (e.g. supervision, pregnancy surveillance) were slightly reduced in the 2^nd^ year of implementation due to the reduced working months and level of effort (%).

**S8 Table G. Estimating the Number of Pregnant Women in a Geographic Area**

|  | Gaibandha district | 1 Million population estimation | Reference |
| --- | --- | --- | --- |
| Population | 2,300,000 | 1,000,000 | [1] |
| Woman of reproductive age (WRA) (15-44 years) | 560,000 | 243,478 |  |
| Fertility rate (birth) (B) per 1000 WRA | 66 | 66 | [2] |
| Abortion rate (A) per 1000 pregnant women | 29 | 29 | [3] |
| Fetal loss rate (death) in 1000 births (D) | 28 | 28 | [4] |
| Pregnant women (in 2015)^1^ | 34,259 | 15,000^2^ | [5] |

1. Number of pregnant women = WRA/1000 * {(B*Pb) + (A*Pa) + (D*Pd)} where P = proportion of the year a woman is pregnant for each pregnancy outcome by month. (Pb: 9 months = .75 Pa: 2 months = .167 Pd: 3 months = .25)
2. We observed 1 CHW served 10 pregnant women enrollment and management over a year in mCARE project. Thus, we estimated 1500 CHWs to be needed for 15,000 pregnant women enrollment and management over a year.

S9 Table H. Standardized costs per 1 million population by study groups for sensitivty analyses

| **Standardized cost per**  **1 million population** | **Comprehensive mCARE** | | | **Basic mCARE** | | |
| --- | --- | --- | --- | --- | --- | --- |
| **Cost** | **Base** | **Low (-20%)** | **High (+20%)** | **Base** | **Low (-20%)** | **High (+20%)** |
| Development and Start up | | | | | | |
| Partnership development | $3,161 | $2,529 | $3,793 | $3,161 | $2,529 | $3,793 |
| Systems development | $10,488 | $8,391 | $12,586 | $10,488 | $8,391 | $12,586 |
| Mobile phones procurement | $77,080 | $61,664 | $92,496 | $77,080 | $61,664 | $92,496 |
| Furniture and equipment | $10,150 | $8,120 | $12,180 | $10,150 | $8,120 | $12,180 |
| System optimization | $736 | $589 | $883 | $736 | $589 | $883 |
| Community outreach | $53,013 | $42,411 | $63,616 | $53,013 | $42,411 | $63,616 |
| Training | $276,603 | $221,283 | $331,924 | $276,603 | $221,283 | $331,924 |
| Implementation (1^st^ year) | | | | | | |
| Supervision | $1,021,816 | $817,453 | $1,226,179 | $1,021,816 | $817,453 | $1,226,179 |
| Census enumeration | $29,767 | $23,814 | $35,721 | $29,767 | $23,814 | $35,721 |
| Pregnancy surveillance | $245,133 | $196,106 | $294,160 | $245,133 | $196,106 | $294,160 |
| Server maintenance | $166,608 | $133,286 | $199,929 | $166,608 | $133,286 | $199,929 |
| SMS reminder | $122,893 | $98,314 | $147,471 | N/A | | |
| Reminder home visits | $47,628 | $38,102 | $57,153 |  |  |  |
| Implementation (2^nd^ year) | | | | | | |
| Supervision | $888,414 | $710,731 | $1,066,097 | $888,414 | $710,731 | $1,066,097 |
| Pregnancy surveillance | $213,130 | $170,504 | $255,756 | $213,130 | $170,504 | $255,756 |
| Server maintenance | $144,857 | $115,885 | $173,828 | $144,857 | $115,885 | $173,828 |
| SMS reminder | $106,849 | $85,479 | $128,218 | N/A | | |
| Reminder home visits | $41,410 | $33,128 | $49,692 |  |  |  |
| Data processing & management | $5,275 | $4,220 | $6,330 | $5,275 | $4,220 | $6,330 |
| **Effectiveness** | **Mean** | **95% uncertainty rate** | | **Mean** | **95% uncertainty rate** | |
| Newborn deaths | 182 | 91 | 273 | 536 | 268 | 804 |

- For probabilistic sensitivity analyses, as the cost estimates are not based on sampled data, we assumed distributions following common standards based on data characteristics. For example, cost parameters are assumed to be a gamma distribution, as the cost distribution is generally right-skewed. For a gamma distribution, standard errors were estimated based on 20% of a point estimate. The number of deaths parameter is assumed as a triangular symmetric distribution as a general standard practice for the value without sample data and evidence of a particular distribution pattern. For triangular symmetric distribution, upper and lower values were from 95% confidence intervals from the outcome samples of the household survey.

**References**

1. Bangladesh Bureau of Statistics. Available from: <http://203.112.218.65:8008/>

2. Global Burden of Disease 2017 Population and Fertility Collaborators. Population and fertility by age and sex for 195 countries and territories, 1950-2017: a systematic analysis for the Global Burden of Disease Study 2017. Lancet, 2018. 392(10159): p. 1995-2051.

3. Benson J, Andersen K, and Samandari G, Reductions in abortion-related mortality following policy reform: evidence from Romania, South Africa and Bangladesh. Reprod Health, 2011. 8: p. 39.

4. Abir T, Agho KE, Ogbo FA, Predictors of stillbirths in Bangladesh: evidence from the 2004-2014 nation-wide household surveys. Glob Health Action, 2017. 10(1): p. 1410048.

5. Centers for Disease Control and Prevention (CDC) Division of Reproductive Health. Estimating the Number of Pregnant Women in a Geographic Area. Available from: <https://www.cdc.gov/reproductivehealth/emergency/pdfs/PregnacyEstimatoBrochure508.pdf>.
